# Bis{6,6′-dimeth­oxy-2,2′-[ethane-1,2-diyl­bis(imino­methyl­ene)]diphenolato(1.5−)-κ^4^ *O*,*N*,*N*′,*O*′}praeseodymium(III)

**DOI:** 10.1107/S1600536809001500

**Published:** 2009-01-17

**Authors:** Hai-Tao Xia, Yu-Fen Liu, Shu-Ping Yang, Da-Qi Wang

**Affiliations:** a School of Chemical Engineering, Huaihai Institute of Technology, Lianyungang 222005, People’s Republic of China; b College of Chemistry and Chemical Engineering, Liaocheng University, Shandong 252059, People’s Republic of China

## Abstract

The title compound, [Pr(C_18_H_22.5_N_2_O_4_)_2_], is isotypic with its Er and Tb analogues. All interatomic distances, angles and the hydrogen bond geometry are very similar for the three structures..

## Related literature

For related structures, see: Liu *et al.* (2007 ▶); Xia *et al.* (2006 ▶). For isotypic structures, see:  Xia *et al.* (2009*a* ▶,*b* ▶).
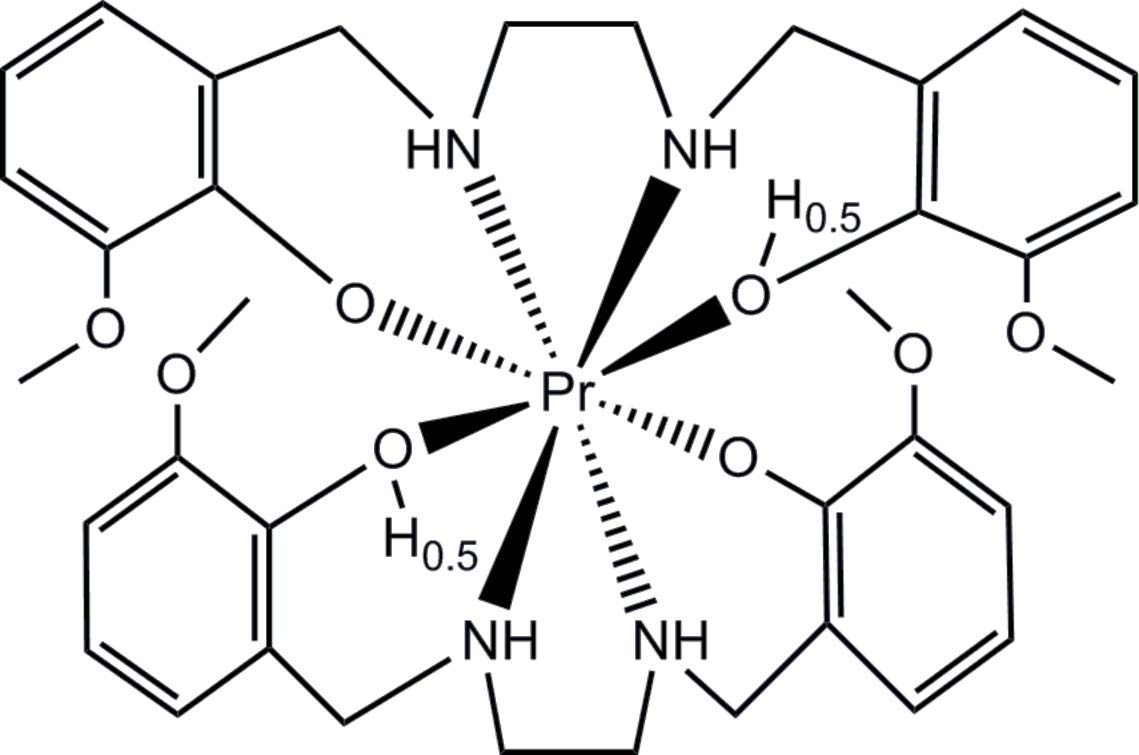


## Experimental

### 

#### Crystal data

[Pr(C_18_H_22.5_N_2_O_4_)_2_]
*M* *_r_* = 802.67
Orthorhombic, 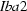

*a* = 21.977 (3) Å
*b* = 11.1616 (12) Å
*c* = 14.1699 (18) Å
*V* = 3475.8 (7) Å^3^
*Z* = 4
Mo *K*α radiation
μ = 1.46 mm^−1^
*T* = 298 (2) K
0.22 × 0.13 × 0.08 mm

#### Data collection

Siemens SMART 1000 CCD area-detector diffractometer
Absorption correction: multi-scan (*SADABS*; Sheldrick, 1996 ▶) *T*_min_ = 0.740, *T*_max_ = 0.892
8635 measured reflections
2878 independent reflections
2230 reflections with *I* > 2σ(*I*)
*R*_int_ = 0.030

#### Refinement

*R*[*F* ^2^ > 2σ(*F* ^2^)] = 0.028
*wR*(*F* ^2^) = 0.070
*S* = 1.07
2878 reflections
222 parameters
1 restraint
H-atom parameters constrained
Δρ_max_ = 0.69 e Å^−3^
Δρ_min_ = −0.37 e Å^−3^
Absolute structure: Flack (1983 ▶), 1271 Friedel pairs
Flack parameter: 0.00 (3)

### 

Data collection: *SMART* (Siemens, 1996 ▶); cell refinement: *SAINT* (Siemens, 1996 ▶); data reduction: *SAINT*; program(s) used to solve structure: *SHELXS97* (Sheldrick, 2008 ▶); program(s) used to refine structure: *SHELXL97* (Sheldrick, 2008 ▶); molecular graphics: *SHELXTL* (Sheldrick, 2008 ▶); software used to prepare material for publication: *SHELXTL*.

## Supplementary Material

Crystal structure: contains datablocks I, global. DOI: 10.1107/S1600536809001500/at2681sup1.cif

Structure factors: contains datablocks I. DOI: 10.1107/S1600536809001500/at2681Isup2.hkl

Additional supplementary materials:  crystallographic information; 3D view; checkCIF report

## Figures and Tables

**Table 1:** Selected bond lengths (Å)

Pr1—O3	2.200 (4)
Pr1—O3^i^	2.200 (4)
Pr1—O1^i^	2.204 (4)
Pr1—O1	2.204 (4)
Pr1—N1^i^	2.619 (4)
Pr1—N1	2.619 (4)
Pr1—N2^i^	2.623 (5)
Pr1—N2	2.623 (5)

Symmetry code: (i) 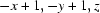.

**Table 2:** Hydrogen-bond geometry (Å, °)

*D*—H⋯*A*	*D*—H	H⋯*A*	*D*⋯*A*	*D*—H⋯*A*
O3—H3*C*⋯O4	0.85	2.12	2.653 (5)	121
N1—H1⋯O4^i^	0.91	2.34	3.229 (6)	167
N2—H2⋯O2^i^	0.91	2.59	3.464 (6)	162

Symmetry code: (i) 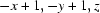.

**Fig. 1.**
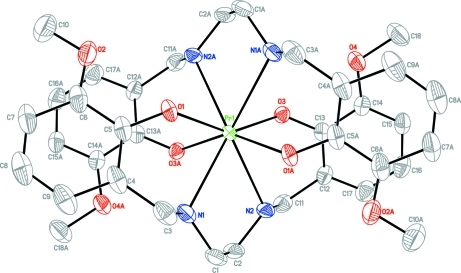
The molecular structure of (I), showing the atom-labelling scheme. Displacement ellipsoids are at the 30% probability level. For clarity, H atoms have been omitted. [Symmetry codes: (A) 1 - x, 1 - y, z].

## supplementary crystallographic information

### Comment

Diamine derivatives are potentially multidentate ligands. we have recently reported the crystal structure (C_18_H_24_O_2_N_4_) (II) (Xia *et al.*, 2006) which is the ligand of the title compound and two complexes [Ce(C_18_H_22_N_2_O_4_)_2_] (III) (Liu *et al.*, 2007), [Er(C_18_H_22.5_N_2_O_4_)_2_] (IV) (Xia *et al.*, 2009). We report here the crystal structure of new rare earth complex , (I).

In the title complex (I), the coordination environment of the Pr atom and coordination modes of (I) ligands to Pr^III^ ion is in agreement with the complexes reported above (Fig. 1). The average bond lengths of between the Praeseodymium center oxygen atoms are 2.202 (4)Å and nitrogen atom are 2.621 (5) Å, longer than the 2.199 (4)Å and shorter than the 2.624 (4)Å of complexes (III), respectively, shorter than the 2.203 (6)Å and longter than the 2.612 (8)Å of complexes (IV), respectively. The dihedral angles between phenyl ring (C4—C9 ring) and antother phenyl ring are 42.03 (16)°(C12—C17 ring), 47.60 (15)°(C4A—C9A ring) and 15.08 (24)°(C12A—C17A ring) [symmetry codes: (A) 1 - *x*, 1 - *y*, *z*].

In (I), the Pr atom is eight-coordinated by four O atoms and four N atoms from two 6,6'-dimethoxy-2,2'-(ethane-1,2-diyldiiminodimethylene)diphenol. The molecules are connected by van der Waals forces, resulting in a three-dimensional network.

### Experimental

A solution of 6,6'-dimethoxy-2,2'-(ethane-1,2-diyldiiminodimethylene) diphenol (0.328 g, 2 mmol) in ethanol (20 ml), and then a solution of Pr(NO_3_)_3_.6H_2_O (0.435 g, 1 mmol) in ethanol (10 ml) was added. The reaction mixture was stirred for 3 h in the air and then filtered. X-ray quality crystals of (I) were obtained by evaporation of an ethanol solution.

### Refinement

All H atoms were located in difference Fourier maps. H atoms bonded to C, O and N atoms were treated as riding atoms, with C—H distances of 0.93 Å (aryl), 0.96 Å (methyl), 0.97Å (methylene) and N—H distances of 0.90 Å (amino), *U*_iso_(H) = 1.2*U*_eq_(aryl, methylene, NH) or 1.5*U*_eq_(C) (methyl or OH). The H3C bonded to O3 is disordered and were refined with the occupancies ties to 0.5.

### Crystal data

**Table:**

[Pr(C_18_H_22.5_N_2_O_4_)_2_]	*F*(000) = 1648
*M**_r_* = 802.67	*D*_x_ = 1.534 Mg m^−^^3^
Orthorhombic, *I**b**a*2	Mo *K*α radiation, λ = 0.71073 Å
Hall symbol: I 2 -2c	Cell parameters from 4426 reflections
*a* = 21.977 (3) Å	θ = 2.9–28.2°
*b* = 11.1616 (12) Å	µ = 1.46 mm^−^^1^
*c* = 14.1699 (18) Å	*T* = 298 K
*V* = 3475.8 (7) Å^3^	Block, brown
*Z* = 4	0.22 × 0.13 × 0.08 mm

### Data collection

**Table:**

Siemens SMART 1000 CCD area-detector diffractometer	2878 independent reflections
Radiation source: fine-focus sealed tube	2230 reflections with *I* > 2σ(*I*)
graphite	*R*_int_ = 0.030
φ and ω scans	θ_max_ = 25.0°, θ_min_ = 1.9°
Absorption correction: multi-scan (*SADABS*; Sheldrick, 1996)	*h* = −26→24
*T*_min_ = 0.740, *T*_max_ = 0.892	*k* = −10→13
8635 measured reflections	*l* = −15→16

### Refinement

**Table:**

Refinement on *F*^2^	Secondary atom site location: difference Fourier map
Least-squares matrix: full	Hydrogen site location: inferred from neighbouring sites
*R*[*F*^2^ > 2σ(*F*^2^)] = 0.028	H-atom parameters constrained
*wR*(*F*^2^) = 0.070	*w* = 1/[σ^2^(*F*_o_^2^) + (0.0246*P*)^2^ + 7.4954*P*] where *P* = (*F*_o_^2^ + 2*F*_c_^2^)/3
*S* = 1.07	(Δ/σ)_max_ = 0.001
2878 reflections	Δρ_max_ = 0.69 e Å^−^^3^
222 parameters	Δρ_min_ = −0.37 e Å^−^^3^
1 restraint	Absolute structure: Flack (1983), 1271 Freidel pairs
Primary atom site location: structure-invariant direct methods	Flack parameter: 0.00 (3)

### Special details

**Table:**

Geometry. All e.s.d.'s (except the e.s.d. in the dihedral angle between two l.s. planes) are estimated using the full covariance matrix. The cell e.s.d.'s are taken into account individually in the estimation of e.s.d.'s in distances, angles and torsion angles; correlations between e.s.d.'s in cell parameters are only used when they are defined by crystal symmetry. An approximate (isotropic) treatment of cell e.s.d.'s is used for estimating e.s.d.'s involving l.s. planes.
Refinement. Refinement of *F*^2^ against ALL reflections. The weighted *R*-factor *wR* and goodness of fit *S* are based on *F*^2^, conventional *R*-factors *R* are based on *F*, with *F* set to zero for negative *F*^2^. The threshold expression of *F*^2^ > σ(*F*^2^) is used only for calculating *R*-factors(gt) *etc*. and is not relevant to the choice of reflections for refinement. *R*-factors based on *F*^2^ are statistically about twice as large as those based on *F*, and *R*- factors based on ALL data will be even larger.

### Fractional atomic coordinates and isotropic or equivalent isotropic displacement parameters (Å^2^)

**Table:**

	*x*	*y*	*z*	*U*_iso_*/*U*_eq_	Occ. (<1)
Pr1	0.5000	0.5000	0.34632 (9)	0.03225 (10)	
N1	0.6052 (2)	0.5730 (5)	0.4112 (4)	0.0494 (13)	
H1	0.6334	0.5339	0.3757	0.059*	
N2	0.5336 (2)	0.7094 (4)	0.2803 (3)	0.0446 (12)	
H2	0.5154	0.7641	0.3186	0.054*	
O1	0.53496 (16)	0.3653 (4)	0.4463 (3)	0.0437 (9)	
O2	0.5617 (2)	0.1320 (4)	0.4296 (4)	0.0719 (14)	
O3	0.43210 (16)	0.5583 (3)	0.2419 (3)	0.0395 (8)	
H3C	0.4017	0.5132	0.2524	0.059*	0.50
O4	0.31350 (17)	0.5867 (4)	0.2690 (3)	0.0547 (11)	
C1	0.6158 (3)	0.7002 (7)	0.3927 (7)	0.063 (2)	
H1A	0.6583	0.7191	0.4037	0.075*	
H1B	0.5914	0.7481	0.4354	0.075*	
C2	0.5994 (3)	0.7299 (6)	0.2921 (6)	0.061 (2)	
H2A	0.6091	0.8129	0.2785	0.074*	
H2B	0.6221	0.6794	0.2490	0.074*	
C3	0.6177 (3)	0.5354 (7)	0.5091 (5)	0.062 (2)	
H3A	0.5823	0.5513	0.5482	0.074*	
H3B	0.6516	0.5809	0.5342	0.074*	
C4	0.6322 (3)	0.4054 (7)	0.5115 (4)	0.0574 (19)	
C5	0.5892 (3)	0.3256 (7)	0.4762 (5)	0.048 (2)	
C6	0.6047 (3)	0.2033 (7)	0.4686 (5)	0.0616 (19)	
C7	0.6621 (3)	0.1636 (9)	0.5003 (6)	0.071 (3)	
H7	0.6725	0.0830	0.4968	0.085*	
C8	0.7014 (4)	0.2440 (10)	0.5356 (6)	0.082 (3)	
H8	0.7394	0.2174	0.5554	0.098*	
C9	0.6885 (3)	0.3609 (9)	0.5435 (5)	0.073 (2)	
H9	0.7168	0.4128	0.5701	0.088*	
C10	0.5795 (4)	0.0130 (7)	0.4047 (8)	0.105 (3)	
H10A	0.6162	0.0159	0.3680	0.157*	
H10B	0.5478	−0.0239	0.3682	0.157*	
H10C	0.5864	−0.0328	0.4610	0.157*	
C11	0.5117 (3)	0.7376 (6)	0.1847 (5)	0.0556 (17)	
H11A	0.5213	0.6721	0.1422	0.067*	
H11B	0.5316	0.8093	0.1616	0.067*	
C12	0.4433 (3)	0.7570 (6)	0.1875 (4)	0.0491 (15)	
C13	0.4073 (3)	0.6640 (6)	0.2217 (5)	0.0414 (17)	
C14	0.3448 (3)	0.6836 (6)	0.2338 (4)	0.0456 (14)	
C15	0.3194 (3)	0.7933 (7)	0.2106 (5)	0.0562 (18)	
H15	0.2779	0.8067	0.2183	0.067*	
C16	0.3569 (4)	0.8827 (7)	0.1757 (6)	0.068 (2)	
H16	0.3399	0.9564	0.1600	0.082*	
C17	0.4164 (4)	0.8664 (6)	0.1639 (5)	0.066 (2)	
H17	0.4403	0.9281	0.1399	0.079*	
C18	0.2529 (3)	0.6065 (8)	0.2982 (6)	0.079 (2)	
H18A	0.2509	0.6785	0.3351	0.119*	
H18B	0.2393	0.5400	0.3356	0.119*	
H18C	0.2272	0.6145	0.2438	0.119*	

### Atomic displacement parameters (Å^2^)

**Table:**

	*U*^11^	*U*^22^	*U*^33^	*U*^12^	*U*^13^	*U*^23^
Pr1	0.02417 (14)	0.03686 (17)	0.03572 (16)	−0.00020 (19)	0.000	0.000
N1	0.030 (2)	0.060 (3)	0.058 (3)	0.001 (2)	−0.002 (2)	−0.025 (3)
N2	0.036 (3)	0.043 (3)	0.055 (3)	−0.009 (2)	0.014 (2)	−0.004 (2)
O1	0.031 (2)	0.059 (3)	0.040 (2)	0.0115 (19)	−0.0036 (17)	0.005 (2)
O2	0.067 (3)	0.057 (3)	0.092 (4)	0.020 (3)	0.017 (3)	0.023 (3)
O3	0.041 (2)	0.037 (2)	0.041 (2)	0.0023 (19)	−0.0060 (19)	0.0011 (18)
O4	0.035 (2)	0.065 (3)	0.064 (3)	0.005 (2)	−0.0034 (19)	0.002 (2)
C1	0.033 (4)	0.068 (5)	0.088 (5)	−0.013 (3)	0.006 (4)	−0.031 (4)
C2	0.045 (4)	0.050 (4)	0.090 (6)	−0.016 (3)	0.020 (4)	−0.011 (4)
C3	0.044 (4)	0.090 (6)	0.052 (4)	0.001 (3)	−0.008 (3)	−0.025 (4)
C4	0.043 (3)	0.090 (6)	0.040 (4)	0.020 (4)	−0.003 (3)	−0.009 (4)
C5	0.042 (4)	0.069 (6)	0.031 (4)	0.017 (4)	0.006 (3)	0.010 (4)
C6	0.054 (4)	0.082 (5)	0.049 (4)	0.023 (4)	0.008 (3)	0.018 (4)
C7	0.054 (5)	0.091 (7)	0.067 (6)	0.030 (5)	0.013 (4)	0.025 (5)
C8	0.062 (5)	0.116 (8)	0.067 (5)	0.032 (5)	−0.008 (4)	0.013 (5)
C9	0.051 (4)	0.116 (7)	0.054 (4)	0.016 (5)	−0.011 (3)	−0.008 (5)
C10	0.100 (6)	0.066 (5)	0.149 (8)	0.021 (5)	0.023 (6)	0.020 (6)
C11	0.064 (5)	0.044 (3)	0.058 (4)	−0.006 (3)	0.022 (3)	0.002 (3)
C12	0.059 (4)	0.043 (4)	0.045 (3)	0.010 (3)	0.006 (3)	0.004 (3)
C13	0.045 (4)	0.046 (4)	0.033 (4)	0.006 (4)	−0.004 (3)	−0.004 (3)
C14	0.051 (3)	0.049 (4)	0.037 (3)	0.009 (3)	−0.005 (3)	−0.003 (3)
C15	0.053 (4)	0.057 (4)	0.058 (5)	0.016 (4)	−0.008 (3)	−0.007 (3)
C16	0.078 (6)	0.051 (5)	0.074 (5)	0.019 (4)	−0.001 (4)	0.010 (4)
C17	0.079 (5)	0.049 (4)	0.071 (5)	0.005 (4)	0.012 (4)	0.012 (4)
C18	0.044 (4)	0.099 (6)	0.095 (5)	−0.001 (4)	0.001 (4)	0.008 (5)

### Geometric parameters (Å, °)

**Table:**

Pr1—O3	2.200 (4)	C3—H3B	0.9700
Pr1—O3^i^	2.200 (4)	C4—C5	1.392 (10)
Pr1—O1^i^	2.204 (4)	C4—C9	1.409 (9)
Pr1—O1	2.204 (4)	C5—C6	1.411 (10)
Pr1—N1^i^	2.619 (4)	C6—C7	1.411 (9)
Pr1—N1	2.619 (4)	C7—C8	1.344 (12)
Pr1—N2^i^	2.623 (5)	C7—H7	0.9300
Pr1—N2	2.623 (5)	C8—C9	1.340 (12)
Pr1—H3C	2.5421	C8—H8	0.9300
N1—C1	1.462 (9)	C9—H9	0.9300
N1—C3	1.475 (8)	C10—H10A	0.9600
N1—H1	0.9100	C10—H10B	0.9600
N2—C11	1.471 (8)	C10—H10C	0.9600
N2—C2	1.472 (8)	C11—C12	1.519 (9)
N2—H2	0.9100	C11—H11A	0.9700
O1—C5	1.340 (7)	C11—H11B	0.9700
O2—C6	1.353 (8)	C12—C13	1.392 (9)
O2—C10	1.428 (9)	C12—C17	1.397 (9)
O3—C13	1.330 (7)	C13—C14	1.402 (8)
O3—H3C	0.8500	C14—C15	1.386 (9)
O4—C14	1.375 (7)	C15—C16	1.386 (11)
O4—C18	1.413 (8)	C15—H15	0.9300
C1—C2	1.508 (9)	C16—C17	1.332 (10)
C1—H1A	0.9700	C16—H16	0.9300
C1—H1B	0.9700	C17—H17	0.9300
C2—H2A	0.9700	C18—H18A	0.9600
C2—H2B	0.9700	C18—H18B	0.9600
C3—C4	1.486 (11)	C18—H18C	0.9600
C3—H3A	0.9700		
			
O3—Pr1—O3^i^	95.47 (19)	N2—C2—H2B	110.1
O3—Pr1—O1^i^	89.65 (13)	C1—C2—H2B	110.1
O3^i^—Pr1—O1^i^	150.52 (13)	H2A—C2—H2B	108.4
O3—Pr1—O1	150.52 (13)	N1—C3—C4	109.8 (5)
O3^i^—Pr1—O1	89.65 (13)	N1—C3—H3A	109.7
O1^i^—Pr1—O1	100.0 (2)	C4—C3—H3A	109.7
O3—Pr1—N1^i^	74.28 (15)	N1—C3—H3B	109.7
O3^i^—Pr1—N1^i^	137.99 (15)	C4—C3—H3B	109.7
O1^i^—Pr1—N1^i^	71.27 (16)	H3A—C3—H3B	108.2
O1—Pr1—N1^i^	82.53 (15)	C5—C4—C9	119.1 (7)
O3—Pr1—N1	137.99 (15)	C5—C4—C3	118.1 (5)
O3^i^—Pr1—N1	74.28 (15)	C9—C4—C3	122.7 (7)
O1^i^—Pr1—N1	82.53 (15)	O1—C5—C4	120.4 (6)
O1—Pr1—N1	71.27 (16)	O1—C5—C6	120.6 (7)
N1^i^—Pr1—N1	138.9 (2)	C4—C5—C6	118.9 (6)
O3—Pr1—N2^i^	80.36 (14)	O2—C6—C7	124.7 (7)
O3^i^—Pr1—N2^i^	71.79 (14)	O2—C6—C5	115.6 (6)
O1^i^—Pr1—N2^i^	137.63 (14)	C7—C6—C5	119.6 (8)
O1—Pr1—N2^i^	73.73 (16)	C8—C7—C6	119.0 (9)
N1^i^—Pr1—N2^i^	66.38 (16)	C8—C7—H7	120.5
N1—Pr1—N2^i^	130.63 (15)	C6—C7—H7	120.5
O3—Pr1—N2	71.79 (14)	C9—C8—C7	123.0 (8)
O3^i^—Pr1—N2	80.36 (14)	C9—C8—H8	118.5
O1^i^—Pr1—N2	73.73 (16)	C7—C8—H8	118.5
O1—Pr1—N2	137.63 (14)	C8—C9—C4	120.2 (8)
N1^i^—Pr1—N2	130.63 (15)	C8—C9—H9	119.9
N1—Pr1—N2	66.38 (16)	C4—C9—H9	119.9
N2^i^—Pr1—N2	138.2 (2)	O2—C10—H10A	109.5
O3—Pr1—H3C	18.9	O2—C10—H10B	109.5
O3^i^—Pr1—H3C	104.0	H10A—C10—H10B	109.5
O1^i^—Pr1—H3C	90.0	O2—C10—H10C	109.5
O1—Pr1—H3C	132.2	H10A—C10—H10C	109.5
N1^i^—Pr1—H3C	56.7	H10B—C10—H10C	109.5
N1—Pr1—H3C	156.4	N2—C11—C12	109.3 (5)
N2^i^—Pr1—H3C	68.0	N2—C11—H11A	109.8
N2—Pr1—H3C	90.0	C12—C11—H11A	109.8
C1—N1—C3	114.5 (6)	N2—C11—H11B	109.8
C1—N1—Pr1	112.3 (4)	C12—C11—H11B	109.8
C3—N1—Pr1	113.9 (4)	H11A—C11—H11B	108.3
C1—N1—H1	105.0	C13—C12—C17	119.7 (6)
C3—N1—H1	105.0	C13—C12—C11	117.7 (5)
Pr1—N1—H1	105.0	C17—C12—C11	122.4 (6)
C11—N2—C2	113.1 (5)	O3—C13—C12	120.2 (6)
C11—N2—Pr1	115.2 (4)	O3—C13—C14	120.9 (6)
C2—N2—Pr1	112.0 (4)	C12—C13—C14	118.9 (6)
C11—N2—H2	105.1	O4—C14—C15	125.4 (6)
C2—N2—H2	105.1	O4—C14—C13	114.3 (5)
Pr1—N2—H2	105.1	C15—C14—C13	120.2 (6)
C5—O1—Pr1	137.6 (4)	C16—C15—C14	118.8 (7)
C6—O2—C10	117.1 (6)	C16—C15—H15	120.6
C13—O3—Pr1	133.2 (4)	C14—C15—H15	120.6
C13—O3—H3C	103.9	C17—C16—C15	122.0 (7)
Pr1—O3—H3C	103.9	C17—C16—H16	119.0
C14—O4—C18	117.0 (5)	C15—C16—H16	119.0
N1—C1—C2	110.2 (6)	C16—C17—C12	120.3 (7)
N1—C1—H1A	109.6	C16—C17—H17	119.8
C2—C1—H1A	109.6	C12—C17—H17	119.8
N1—C1—H1B	109.6	O4—C18—H18A	109.5
C2—C1—H1B	109.6	O4—C18—H18B	109.5
H1A—C1—H1B	108.1	H18A—C18—H18B	109.5
N2—C2—C1	108.0 (6)	O4—C18—H18C	109.5
N2—C2—H2A	110.1	H18A—C18—H18C	109.5
C1—C2—H2A	110.1	H18B—C18—H18C	109.5
			
O3—Pr1—N1—C1	19.9 (5)	N1—C1—C2—N2	63.9 (7)
O3^i^—Pr1—N1—C1	100.5 (5)	C1—N1—C3—C4	−155.2 (5)
O1^i^—Pr1—N1—C1	−61.2 (4)	Pr1—N1—C3—C4	73.6 (5)
O1—Pr1—N1—C1	−164.4 (5)	N1—C3—C4—C5	−57.9 (8)
N1^i^—Pr1—N1—C1	−111.1 (5)	N1—C3—C4—C9	118.9 (6)
N2^i^—Pr1—N1—C1	148.5 (4)	Pr1—O1—C5—C4	53.9 (9)
N2—Pr1—N1—C1	14.3 (4)	Pr1—O1—C5—C6	−123.3 (6)
O3—Pr1—N1—C3	152.2 (4)	C9—C4—C5—O1	179.2 (6)
O3^i^—Pr1—N1—C3	−127.3 (4)	C3—C4—C5—O1	−3.9 (9)
O1^i^—Pr1—N1—C3	71.1 (4)	C9—C4—C5—C6	−3.5 (10)
O1—Pr1—N1—C3	−32.2 (4)	C3—C4—C5—C6	173.4 (6)
N1^i^—Pr1—N1—C3	21.1 (4)	C10—O2—C6—C7	−10.9 (10)
N2^i^—Pr1—N1—C3	−79.3 (4)	C10—O2—C6—C5	168.7 (7)
N2—Pr1—N1—C3	146.6 (4)	O1—C5—C6—O2	0.3 (9)
O3—Pr1—N2—C11	−26.1 (4)	C4—C5—C6—O2	−177.0 (6)
O3^i^—Pr1—N2—C11	72.9 (4)	O1—C5—C6—C7	179.9 (6)
O1^i^—Pr1—N2—C11	−121.3 (4)	C4—C5—C6—C7	2.6 (10)
O1—Pr1—N2—C11	151.7 (3)	O2—C6—C7—C8	178.3 (7)
N1^i^—Pr1—N2—C11	−75.0 (4)	C5—C6—C7—C8	−1.2 (11)
N1—Pr1—N2—C11	149.9 (4)	C6—C7—C8—C9	0.9 (13)
N2^i^—Pr1—N2—C11	24.7 (3)	C7—C8—C9—C4	−1.9 (13)
O3—Pr1—N2—C2	−157.4 (5)	C5—C4—C9—C8	3.2 (11)
O3^i^—Pr1—N2—C2	−58.3 (4)	C3—C4—C9—C8	−173.5 (7)
O1^i^—Pr1—N2—C2	107.5 (5)	C2—N2—C11—C12	−160.1 (5)
O1—Pr1—N2—C2	20.5 (5)	Pr1—N2—C11—C12	69.3 (5)
N1^i^—Pr1—N2—C2	153.8 (4)	N2—C11—C12—C13	−57.6 (7)
N1—Pr1—N2—C2	18.7 (4)	N2—C11—C12—C17	117.5 (7)
N2^i^—Pr1—N2—C2	−106.6 (4)	Pr1—O3—C13—C12	64.1 (8)
O3—Pr1—O1—C5	144.6 (6)	Pr1—O3—C13—C14	−116.3 (6)
O3^i^—Pr1—O1—C5	44.1 (6)	C17—C12—C13—O3	178.1 (6)
O1^i^—Pr1—O1—C5	−107.9 (6)	C11—C12—C13—O3	−6.7 (9)
N1^i^—Pr1—O1—C5	−177.3 (6)	C17—C12—C13—C14	−1.5 (10)
N1—Pr1—O1—C5	−29.4 (6)	C11—C12—C13—C14	173.8 (5)
N2^i^—Pr1—O1—C5	115.2 (6)	C18—O4—C14—C15	−11.0 (9)
N2—Pr1—O1—C5	−31.1 (7)	C18—O4—C14—C13	169.3 (6)
O3^i^—Pr1—O3—C13	−117.1 (6)	O3—C13—C14—O4	1.1 (9)
O1^i^—Pr1—O3—C13	33.8 (5)	C12—C13—C14—O4	−179.3 (5)
O1—Pr1—O3—C13	143.9 (5)	O3—C13—C14—C15	−178.6 (6)
N1^i^—Pr1—O3—C13	104.4 (6)	C12—C13—C14—C15	0.9 (10)
N1—Pr1—O3—C13	−44.6 (6)	O4—C14—C15—C16	−179.9 (6)
N2^i^—Pr1—O3—C13	172.5 (6)	C13—C14—C15—C16	−0.2 (11)
N2—Pr1—O3—C13	−39.1 (5)	C14—C15—C16—C17	0.0 (12)
C3—N1—C1—C2	−177.7 (5)	C15—C16—C17—C12	−0.5 (12)
Pr1—N1—C1—C2	−45.7 (6)	C13—C12—C17—C16	1.2 (11)
C11—N2—C2—C1	178.9 (6)	C11—C12—C17—C16	−173.8 (7)
Pr1—N2—C2—C1	−48.8 (6)		

Symmetry codes: (i) −*x*+1, −*y*+1, *z*.

### Hydrogen-bond geometry (Å, °)

**Table:**

*D*—H···*A*	*D*—H	H···*A*	*D*···*A*	*D*—H···*A*
O3—H3C···O4	0.85	2.12	2.653 (5)	121
N1—H1···O4^i^	0.91	2.34	3.229 (6)	167
N2—H2···O2^i^	0.91	2.59	3.464 (6)	162

Symmetry codes: (i) −*x*+1, −*y*+1, *z*.
